# Fish Acquisition and Consumption in the African Great Lakes Region through a Food Environment Lens: A Scoping Review

**DOI:** 10.3390/nu13072408

**Published:** 2021-07-14

**Authors:** Julia de Bruyn, Joshua Wesana, Stuart W. Bunting, Shakuntala H. Thilsted, Philippa J. Cohen

**Affiliations:** 1 Natural Resources Institute, University of Greenwich, Chatham Maritime, Kent ME44TB, UK; j.wesana@greenwich.ac.uk (J.W.); s.w.bunting@greenwich.ac.uk (S.W.B.); 2 Melbourne Sustainable Society Institute, University of Melbourne, Parkville, VIC 3010, Australia; 3 WorldFish, Jalan Batu Maung, Batu Maung, Bayan Lepas 11960, Penang, Malaysia; S.Thilsted@cgiar.org (S.H.T.); P.Cohen@cgiar.org (P.J.C.)

**Keywords:** fish, diet, nutrition, food system, food environment, African Great Lakes Region, Sub-Saharan Africa

## Abstract

Effective actions for the fishery and aquaculture sectors to contribute toward improving nutrition rely on an understanding of the factors influencing fish intake, particularly amongst vulnerable populations. This scoping review synthesises evidence from 33 studies in the African Great Lakes Region to examine the influence of food environments on fish acquisition and consumption. We identified only two studies that explicitly applied a food environment framework and none that linked policy conditions with the contribution of fish to diets. Economic access to fish was represented in the largest number of included studies (21 studies), followed by preferences, acceptability and desirability of fish (17 studies) and availability and physical access (14 studies). Positive perceptions of taste and low cost, relative to other animal-source foods, were drivers of fish purchases in many settings; however, limited physical and economic access were frequently identified as preventing optimal intake. In lakeside communities, fish were increasingly directed toward external markets which reduced the availability and affordability of fish for local households. Few studies considered intra-household variations in fish access according to age, gender or physiological status, which represents an important knowledge gap. There is also scope for future research on seasonal influences on fish access and the design and rigorous evaluation of programmes and policies that address one or more constraints of availability, cost, convenience and preferences.

## 1. Introduction

Achieving sustainable, equitable and large-scale improvements in food and nutrition security is amongst the greatest global challenges. Progress to date has been uneven, with recent estimates that one in three people (33.6%) in Eastern Africa is chronically undernourished [1]. Of 41 countries in which high levels of three forms of malnutrition (i.e., child stunting, anaemia in women of reproductive age and overweight amongst women) coexist, 30 (73%) are in Africa [2]. There is growing interest in the concept of the food system to identify a broad range of entry points to address these nutritional problems [3]. This includes examining the influence of the food environment: the spectrum of external and personal factors that mediate food acquisition and consumption [4,5]. A food systems approach encompasses system-level drivers of governance and sustainability and also focuses attention on the roles and responsibilities that different actors play in ensuring that food is available, accessible and acceptable to all [6].

International development agencies, researchers and funders increasingly seek to position specific commodities or sectors in relation to wider food systems. There is value in operationalising food systems frameworks within a “whole-of-diet” approach [7]: to identify opportunities for combinations of different nutrient-rich food items to meet nutrient gaps and contribute to healthy, sustainable diets. The nutrition and sustainability attributes of fish [8,9] make it of high interest in food system transformations, yet fish production and capture has been neglected in many food systems policies and discussions to date. The Global Panel on Agriculture and Food Systems for Nutrition has invited greater reflection on how the expansion of aquaculture in low- and middle-income countries may positively affect food and nutrition security, economic growth and employment and the environment [10].

Fish provide a rich source of bioavailable micronutrients, long-chain polyunsaturated fatty acids (PUFAs) and high-quality protein and have the potential to increase the nutritional adequacy of diets based on staple foods [11]. In many settings, the burden of micronutrient deficiencies falls disproportionately on young children and on pregnant and breastfeeding women, due to the combined influence of heightened nutrient requirements per unit of body mass, relative to other age groups, and lower social status, affecting intra-household food allocation. Daily intakes of up to 100 g of fish are included in the planetary health reference diet proposed by the EAT Lancet Commission, with whole small fish highlighted as a crucial source of key micronutrients for these vulnerable populations [12]. A paucity of robust data to quantify catches from subsistence and small-scale fishing operations, yields from small-scale aquaculture systems and informal fish trade masks the magnitude and diversity of fish within local, national and regional food systems [13].

While capture fisheries will continue to provide the majority of fish to African diets, fish imports and aquaculture are expected to grow and may narrow the fish supply gaps in the Eastern African sub-region [14]. Foresight models that project fish supply and demand trends indicate that a range of strategies (i.e., including and beyond increasing supply) will be needed in order for fish to contribute more substantially to food and nutrition security in Africa: growing sustainable aquaculture, reducing post-harvest losses, maintaining wild supplies accessed through small-scale fisheries and developing new products and distribution strategies to ensure that fish reach those most affected by nutritionally inadequate diets [15].

Beyond national fish supply targets, there is a need to consider the temporal, geographic, socioeconomic, cultural, gender and age-related factors that influence the acquisition and consumption of fish and, ultimately, its relative contribution to nutrition and health. Food environment frameworks have not typically been linked to a single commodity, but to consider the ways in which surroundings and circumstances influence the breadth of dietary options and choices which exist within a given setting. Their use has been most extensive in high-income countries, historically associated with the “obesogenic” nature of an environment [16,17,18]. We suggest that there is also value in applying food environment concepts to examine the factors that enable or inhibit access to specific nutritious foods, such as fish, by nutritionally vulnerable populations and individuals to inform programmes and policies with greater impact.

The African Great Lakes are amongst the most important freshwater ecosystems globally: rich in aquatic biodiversity [19] and supporting livelihoods and diets [20]. The Great Lakes system has a combined catchment area of more than 850,000 km^2^ [21] and includes Lake Victoria, Lake Tanganyika and Lake Malawi, as well as smaller natural lakes and constructed reservoirs, including Lakes Turkana, Albert, Kyoga, Kivu, Edward, George and Naivasha [22]. The African Great Lakes Region (AGLR) is variably defined but for the purpose of this article is taken to include Burundi, the Democratic Republic of Congo, Kenya, Malawi, Rwanda, Tanzania, Uganda and Zambia.

This paper presents findings of a scoping study conducted to identify factors that mediate fish intake by populations in the AGLR. Whilst previous reviews have examined production trends, supply constraints and linkages with food and nutrition security [14,23,24], we have applied a food environment lens to collate and appraise primary evidence of the factors which promote or inhibit fish intake. Our review has sought to identify knowledge gaps to be addressed through future food systems research and to highlight findings of relevance to programmes, policies and investment decisions in the region.

## 2. Materials and Methods

This study follows PRISMA-ScR guidelines to review existing literature, consider the scope and types of knowledge available and identify and analyse gaps in the evidence base [25,26]. Our primary research question was: What is the available evidence of factors within the food environment influencing fish acquisition and consumption by populations in the AGLR? Search syntax was developed with the aim of capturing any paper referring to the acquisition or consumption of fish by populations in the eight countries, or associated with the six major lakes, of the region:

Fish* AND (food OR diet* OR nutri* OR consum* OR intake OR eat* OR purchas* OR buy* OR bought OR trade OR acqui*) AND (Burundi OR “Democratic Republic of the Congo” OR “Democratic Republic of Congo” OR DRC OR Kenya OR Malawi OR Rwanda OR Tanzania OR Uganda OR Zambia OR (Lake* AND (Tanganyika OR Victoria OR Albert OR Edward OR Kivu OR Malawi))).

This search strategy was intentionally broad, rather than being informed *a priori* by specific themes or dimensions of a food environment. The search was conducted in April 2021, using Web of Science and Scopus. Returned articles were exported to EndNote reference manager for removal of duplicates, title and abstract screening, article examination and data extraction. This process was conducted by two researchers, with consensus reached on the inclusion and exclusion of articles. Our review considers both quantitative and qualitative research findings. All study designs were eligible for inclusion, including case studies, cross-sectional studies and impact evaluations. We included studies published since 2010 in peer-reviewed journals in English. Charting of data involved extraction of details on research design, geographic location, sample size, population group, relevant methods and indicators, key findings, authors and year of publication (Supplementary Material, Table S1).

**Figure 1:**
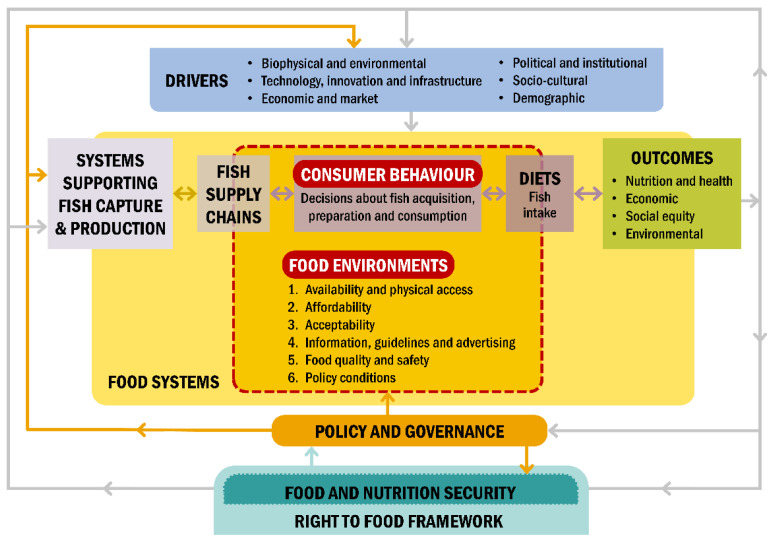
An adapted version of a food systems framework [3], positioning fish supply chains and the contribution of fish to diets within the wider food system. This review collates evidence of aspects of the food environment which influence fish acquisition and consumption by populations in the African Great Lakes Region.

We collated evidence of determinants of fish acquisition and consumption, based on six food environment dimensions presented in the food system framework of the High Level Panel of Experts on Food Security and Nutrition [3]: (1) availability and physical access; (2) prices and affordability; (3) preferences, desirability and acceptability; (4) food quality and safety; (5) information, guidelines and advertising; and (6) policy conditions (Figure 1). This draws on concepts from the Agriculture, Nutrition and Health Academy Food Environment Working Group’s framework [4] but avoids the separation of “personal” and “external” domains, by which individual- or household-level factors are distinguished from wider contextual factors (e.g., food affordability vs. food prices).

## 3. Results

**Figure 2:**
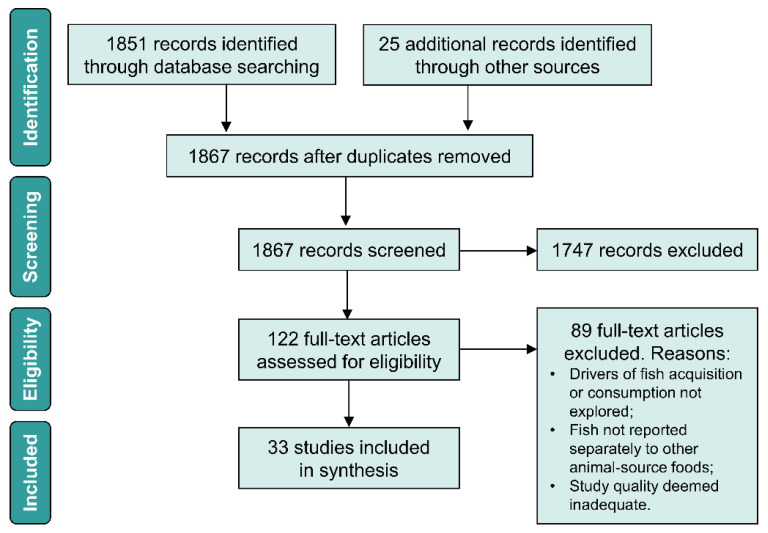
Studies reviewed and excluded at each stage.

As shown in Figure 2, our search returned 1851 references, and 25 additional references were identified through alternative sources. Screening of titles and abstracts led to shortlisting 122 studies for further consideration. Thirty-three met criteria for inclusion in this scoping review. The decision to exclude articles based on review of their full text was most commonly due to a lack of relevant information on drivers of dietary or food procurement behaviours, and less commonly due to fish being grouped together with other animal-source foods in a manner which prevented meaningful conclusions for this review.

**Figure 3:**
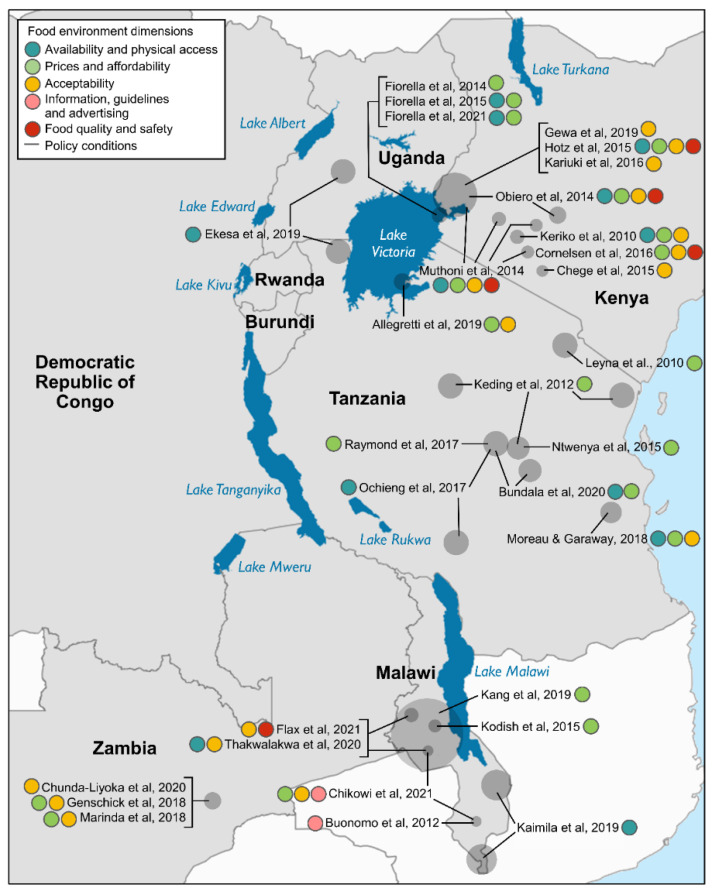
Map of the African Great Lakes Region showing the approximate location of studies in this review which report on factors influencing fish acquisition and consumption. Colours indicate different dimensions of the food environment.

Table 1 and Figure 3 outline the distribution of included studies that present findings associated with five of the six dimensions of the food environment. No relevant studies from Burundi, Rwanda or the Democratic Republic of Congo, or studies evaluating the influence of policy conditions on fish acquisition or consumption in the AGLR, were identified by our search. Findings relating to economic access to fish were represented in the largest number of included studies (21 studies), followed by preferences, acceptability and desirability of fish (17 studies) and fish availability and physical access (14 studies).

### 3.1. Availability and Physical Access

Availability and physical access to fish were most commonly described in terms of proximity to water bodies. Inclusion of small fish as part of a common meal for young children in Bukoba District in Tanzania, but not in Kiboga District in Uganda, was attributed to the former communities being close to Lake Victoria [27]. Similarly, high fish consumption by Tanzanian women [28] and Malawian children [29] in lakeside communities was linked to their easy physical access to fish. Geostatistical modelling has identified a lower risk of selenium deficiency in populations close to Lake Malawi, with authors linking this to these communities’ greater physical access to fish [30].

We found only one study that examined direct participation in fishing activities as a predictor of household fish consumption. Fishing households consumed fish more often and in larger quantities compared to non-fishing households, regardless of wealth, but this effect was greatest amongst poor households [31]. In the early stages of the COVID-19 pandemic in June 2021, phone interviews with members of Kenyan communities by Lake Victoria revealed fishers were more likely to sell than consume fish [32]. The authors suggested that movement restrictions and concerns about COVID-19 may limit fishing activities, leading fishers to target and sell high-value fish in order to purchase less expensive fish for home consumption, if they consume fish at all.

Five studies examined fish availability and access in the context of markets. In Tanzania, a twofold higher likelihood of fish consumption in Morogoro Region, compared to Dodoma Region, was attributed to greater market access [33]. A study of drivers of food consumption by women and young children in central Malawi found that shorter travel time to the nearest market or shop predicted higher fish consumption in the rainy season, whilst living in an urban area, compared to a rural area, was linked to higher fish consumption in the dry season [34]. On the shores of Lake Naivasha, household access to fish through local markets was limited by competition with large lakeside hotels and export to Nairobi, with respondents indicating they would consume fish more frequently if it were more available [35]. Markets were identified as the primary source of both wild-caught and farmed fish in both urban [36] and rural [37] settings in Kenya.

Two studies considered the influence of gender on fish access. Qualitative research to characterise the dietary practices of men in central and southern Tanzania highlighted that fish was amongst foods commonly accessed outside the home, from restaurants or street vendors during the course of the day, but uncommon for other family members to consume in household meals [38]. Near Lake Victoria, relationships with fishers mediate women’s access to fish for trade or home consumption [39]. Women were found to be more likely to participate in transactional sexual (“fish-for-sex”) relationships in the absence of marital relationships or other family ties with fishers, and when fish catches are low.

### 3.2. Prices and Affordability

Economic access was the most studied dimension of the food environment within studies included in this review. The low cost of fish, relative to meat, was identified as a driver of fish consumption in eastern and western Kenya [37], southern Malawi [29] and lakeside communities in Tanzania [27]; however, the high cost of animal-source foods in general, relative to plant-source foods, has been recognised to limit consumption of fish and other animal-source foods by vulnerable households [40]. In Tanzanian communities with high levels of poultry-keeping, fish were more commonly eaten than chicken meat or eggs, with income from poultry-keeping used to purchase fish from markets [33]. Recommendations for optimised diets to meet nutrient requirements of children under two years of age in central Tanzania proposed a substantial increase in the intake of small fish; however, this translated to a twofold increase in the overall cost of diet [41].

Price was the main barrier to fish consumption in Nairobi [42], where demand for fish was the most sensitive to price changes of all animal-source foods, as well as in communities around Lake Naivasha [35]. Communities on Ukerewe Island in Lake Victoria described the development of fish supply chains catering to international markets as having come at the expense of fish consumption by local families, with large fish such as Nile perch unaffordable to many households [43]. In Blantyre and Lilongwe, the effect of price changes on fish purchases varied between species and modes of preparation (i.e., fresh, smoked or dried), with a one percent price rise associated with a reduction of between 22% and 51% in quantities of fish purchased [44].

Caregivers of young children in Malawi identified fish and meat as “rare foods”, consumed infrequently due to their high price [45]. In contrast, price was reported as amongst the least relevant factors influencing consumption of tilapia and African catfish (*Clarias gariepinus*), compared to availability, perceived quality and taste preferences, in rural [46] and urban [36] settings in Kenya. The influence of price on the frequency of fish consumption and selection of particular fish species did not vary across socioeconomic strata of households in Lusaka [47].

Analysis of nationally representative data from Malawi revealed a higher probability of fish consumption to be associated with greater household wealth, as measured by expenditure quintiles [30]. Poor households in rural Tanzania ate fish around half as often as wealthier households; however, small fish were accessible to most, in varying quantities according to households’ financial capacity [31]. In Lusaka, greater wealth was associated with more frequent fish consumption, a greater diversity of fish species, and larger-sized fresh fish [47,48]. Higher consumption of tilapia was identified amongst the highest of four socioeconomic strata, while dried small fish were more common amongst the lowest two strata. Households in the poorest group accessed an average of five fish species, compared to 11 species by those in the wealthiest group [47]; however, there was no significant variation in the frequency of small fish consumption (in fresh, dried or smoked forms) according to socioeconomic status [48].

In a Malawian study that found demand for greater quantities of tilapia to be associated with higher levels of education of household food decision makers, the authors suggested that this reflected higher disposable income [44]. Men and women in Kenyan communities by Lake Victoria indicated income availability may not be sufficient to access fish for sale (for on-selling) or home consumption, with a sexual relationship with a fisherman often a necessary “extra-monetary” strategy for women to make a purchase [39].

In rural Malawi, severe food insecurity was associated with a threefold lower probability of consuming fish or meat for pregnant women and a fivefold lower probability for breastfeeding women [49]; however, the relative affordability of different animal-source foods was not examined. Food insecurity has also been associated with lower fish consumption in Morogoro Region of Tanzania [50], but with no difference in fish consumption near Mount Kilimanjaro [51]. In another Tanzanian study, fish was consumed by more than three quarters of women with medium or high dietary diversity, compared to only one third of those with low diversity [52].

### 3.3. Preferences, Desirability and Acceptability

Studies reporting on preferences, desirability and acceptability of consuming fish considered the influence of sensory attributes (14 studies), cultural factors (8 studies) and convenience (2 studies). Around three quarters of low-income urban households in Nairobi [42] and Lusaka [47] reported taste as the main reason for consuming fish. The latter study reported no significant difference in the influence of taste on fish purchases between households of different wealth strata; however, tilapia was the most common fish for twice as many households in the highest wealth quartile compared to the lowest quartile (49% and 22%, respectively). The authors suggested that while a majority of households reported their fish purchases to be driven by taste, this is likely to reflect familiarity and food habits, with poorer households’ frequent acquisition of low-cost small fish informing their expressed “preferences”.

Findings from Malawi revealed maternal taste preferences for fish (based on a hedonic preference scale) predicted higher fish consumption during the dry season [34], and taste preferences had a greater influence on the choice of tilapia products than the quantities of tilapia purchased [44]. Of two Kenyan studies with a focus on Nile tilapia and African catfish, one found taste to be the primary reason for fish consumption for around one in three (35%) urban respondents [36], while the other reported both taste and smell to have a small influence on fish purchases by rural consumers [46]. The latter study identified fresh fish to be widely preferred to smoked fish but reported marked differences in specific taste preferences between two study sites [46]. Odour was mentioned as a deterrent to consuming fish by a small proportion of respondents in one study close to Lake Naivasha [35] and another in Nairobi [42].

Tanzanian women living by Lake Ruwe reported a preference for oily fish to save money on cooking oil and fish that produced a tasty broth when boiled, suitable for young children [31]. Women in this study also preferred fish that could be cut into large fillets, without many bones, but indicated frying could be used to soften unpalatable tiny bones and small fish eaten whole. In Kenya and Tanzania, caregivers of children 6–23 months of age generally associated animal-source foods with positive attributes for children; however, negative perceptions of consuming fish included fears that children might choke on bones [53]. This led some caregivers to completely avoid giving fish to young children, whereas other cares took extra care in separating bones. Elsewhere in Kenya, a small number of caregivers raised concerns that whole small fish were not appropriate for young children due to chewing difficulties, which was a reason for infrequent consumption by children under 12 months of age [37].

In low-income urban households in Zambia, a large majority of women (90%) reported no change in fish consumption during their pregnancy or lactation [48]. Amongst women from rural and urban communities accessing a district hospital in western Kenya, 6% reported craving fish and 11% described an aversion to fish during pregnancy [54]. Small fish was the most common of all foods avoided during pregnancy, by 15% of women, compared to avoidance of large fish by 11% and beef, milk and eggs by 13%, 5% and 4%, respectively. In a consumer acceptance study in communities bordering Lake Victoria, a novel fish-enhanced snack was rated favourably by over 85% of pregnant women for taste, odour, texture and colour [55].

Fish was named as a special food that should be consumed during pregnancy by women attending antenatal clinics in Lusaka, though women did not convey knowledge about its significance for specific maternal, foetal or infant outcomes [56]. In contrast, taboos preventing fish consumption were identified in three Kenyan studies, relating to a perceived similarity between the skin of fish and reptiles [35], a concern that fish consumption during pregnancy could cause breastfeeding difficulties [57] and a widespread belief within Maasai culture that aquatic animals are unsuitable for human consumption [58].

The role of fish in diets was one of the few differences in food cultures between two rural study sites in Kenya, whereby small fish were considered a core food for young children and their households in communities close to Lake Victoria, but not in a drought-prone area of Eastern Province [37]. Ethnographic research in fishing communities of Ukerewe Island revealed widespread consensus that “fish must be on the table”, regardless of what else might be eaten [43]. Commoditisation of fish was associated with a sense of loss and implicated in a shift in social dynamics from a time when fish was eaten regularly, in large quantities, and shared with neighbours, to an occasional food consumed within the home.

### 3.4. Food Quality and Safety

Of five studies that included findings related to food quality, three related to consumers’ understanding of health and nutritional attributes of fish. In areas of central and western Kenya with large numbers of commercial fish farmers, consumers reported perceptions of “good quality” to be the main driver of fish purchases, while very few were guided by health attributes or nutritional value [46]. Women in central Malawi also named quality as one of the top factors driving food purchases—in relation to all types of food, but particularly for fish, fruits and vegetables [59]. Participants described quality in terms of the appearance and freshness of food items and did not link this with food safety.

Caregivers in rural Kenyan communities ranked whole small fish lower than chicken in terms of perceived health attributes for children [37]. In contrast, nutritional value was an important driver of fish consumption for two thirds of study participants in low-income areas of Nairobi [42], and 40% of respondents from five urban sites in Kenya considered fish to be a healthy food (from 48% in Nairobi to 16% in Kisumu) [36]. The same study revealed some negative perceptions of farmed fish, relating to health concerns about the use of chemicals and genetically modified feed ingredients. We found no studies on food safety as a consideration for fish acquisition and consumption.

### 3.5. Information, Guidelines and Advertising

One study amongst HIV-exposed children in Blantyre, Malawi, reported a significant increase in fish consumption (22% to 47%) after a six-month nutrition support programme, which included nutrition education sessions that encouraged enrichment of infant porridges with dried or ground small fish [60]. In Blantyre and Lilongwe, consumers who accessed information on fish product availability and market prices generally consumed more tilapia products than those who did not [44]. We found no other studies linking information, guidelines or advertising with fish access or consumption.

## 4. Discussion

### 4.1. Key Findings and Evidence Gaps

Concepts and frameworks relating to the food environment are gaining prominence in the Global South; however, to date, studies in which these have been explicitly applied have largely been conducted in upper middle-income countries [61]. In this review, we have collated and appraised evidence of the extent to which aspects of the food environment—the interface between consumers and the wider food system—mediate fish acquisition and consumption. We found that only two included studies have explicitly applied a food environment framework within their research [34,59]. We examined 33 papers reporting on factors associated with fish acquisition and consumption by populations in the AGLR, which we aligned with five of six dimensions of the food environment [3]. We did not identify any studies linking policy conditions with the contribution of fish to diets in the region. Included studies were unevenly distributed, with a predominance of research focused on Kenya, Tanzania and Malawi and comparatively few studies in Uganda, despite also sharing Lake Victoria’s waters and shorelines.

Availability and physical access emerge as key determinants of fish consumption, though often as an inference based on the location of study sites, rather than direct assessment of households’ engagement in fishing activities or opportunities for market purchases. This corresponds with several studies’ findings that a higher likelihood of children consuming fish is associated with proximity to coastal areas or inland water bodies [62,63,64]. Economic access was examined in almost two thirds of included studies. Affordability relative to other animal-source foods and the opportunity for households to purchase small quantities of fresh fish or fish-based products according to their financial means are key advantages of fish, as has been highlighted elsewhere [65,66,67]. Despite this, diets containing optimal amounts of fish to meet nutrient requirements were shown to substantially increase the overall cost of diet in populations largely reliant on plant-source foods [41]. Complementary strategies to increase affordability are necessary to ensure that fish contributes to the nutrition and health of more people, particularly those that experience economic and nutritional vulnerability.

The influence of consumer preferences on fish purchases varied between settings, with findings suggesting that product traits such as taste may influence food acquisition to a greater extent in urban areas than in rural areas. In the single consumer acceptance study in this review, consumers responded favourably to a novel processed fish product, which could improve access to a nutrient-rich food with a prolonged shelf-life [55]. Findings aligned with the “acceptability” dimension of the food environment were drawn largely from studies that examined attitudes towards fish, overall or according to species or mode of preparation, rather than broader assessments of the extent to which preferences influence food purchasing decisions. One included study, published in 2020, is identified by its authors as being the first to empirically measure how taste preferences, a key indicator of desirability and acceptability of food, predict consumption behaviours in Sub-Saharan Africa [34].

While most studies included in this review were cross-sectional, many described temporal changes in fish access or intake—including between dry and rainy seasons [34], following the provision of nutritional counselling and education [60], associated with the development of fish supply chains [43] and in association with the COVID-19 pandemic [32]. Temporal variation in fish consumption is likely to accompany changes in both physical access, due to the seasonal nature of catches and fishing restrictions, and economic access, due to fluctuations in market prices and consumers’ purchasing power (e.g., amongst smallholder farming households reliant on income from an annual or biannual crop harvest). The dynamic nature of fish access, and diets more broadly, emphasises that there will be periods when interventions that ensure access to fish may be most impactful.

Intra-household food and nutrient distribution varies between settings, with some included studies describing culturally sanctioned patterns that favour adult men and younger household members over women, and others finding no consistent pattern [68,69,70]. Most studies in this review reported on fish access at a household level, with few considering how opportunities to consume fish may differ between household members, according to age, gender or physiological status. Eight studies reported on practices and perceptions relating to fish consumption by children, highlighting fish to be a common food for children when not constrained by physical and economic access. Caregivers generally associated fish with positive attributes for children, although some expressed concerns about the appropriateness of fish for infants. Evidence of differential access to fish by men and women was only found in a single study which highlighted opportunities for men, but not women, to acquire and consume fish outside of meals prepared at home [38].

### 4.2. Potential Actions to Enable Greater Fish Access and Intake

Our review has highlighted substantial variation in food environments, even on the shores of the same lake. There is a need for context-specific assessments to understand the social, ecological and economic characteristics of fisheries, and different trajectories and trade-offs with aquaculture development, in the AGLR [71], and for policies to support a diversity of fish species, with a particular focus on micronutrient-rich small fish.

Low availability and unaffordability of fish was reported to limit fish intake in ten included studies. The fisheries and aquaculture sectors have tended to emphasise the need to increase production volumes; however, concurrent actions to improve safety and efficiency of fish processing also have the potential to increase the contribution of fish to human diets [72]. Enhanced capture fisheries management, storage and processing innovations that reduce waste and loss, fish-based products that enable nutrient retention with an extended shelf life and harmonisation of cross-border trade arrangements could address identified constraints in physical and economic access to quality fish products by consumers, including through wider and more efficient intraregional fish trade [73,74].

Geographic lengthening of value chains, combined with appropriate processing technologies and market infrastructure, has the potential to enable consumption by greater numbers of people in areas distant to fisheries and aquaculture production systems, including nutritionally vulnerable groups and those in urban food deserts (characterised by limited access to healthy, nutritious foods based on availability or price) [23,75]. Such developments are needed to ensure that potential increases in fish production from well-managed fisheries [76] are available for direct human consumption, rather than diverted to animal feed and subsequent animal-source food production, possibly with reduced nutrient density and greater environmental impacts [77,78].

Diets that meet global guidelines to maximise both human and planetary health [12] may be physically or economically inaccessible to poor and vulnerable consumers in Sub-Saharan Africa, where the cost of such a diet equates to around three quarters (73%) of mean daily per capita household income [79]. While national food price data in Zambia show the cost of food has generally declined over time, nutrient-rich foods have become more expensive relative to staple foods [80].

Fish-enriched snack foods or supplements, which take advantage of the peak season for fish capture, may present opportunities to improve the diets of undernourished and nutritionally vulnerable population groups within the region, through convenient and targeted delivery of nutrients at a relatively low cost [81,82]. Behaviour change communication strategies are likely to be central to improving perceptions of small fish as a nutritionally important food [11], highlighting its relevance for particular household members, including women and children, and addressing parental concerns about palatability and appropriateness of texture for young children. These can be supported by explicit nutritional messaging from government agencies, which differentiates small and large fish from alternative protein sources and promotes their micronutrient content in food-based dietary guidelines and recommendations for maternal diets and infant and young child feeding practices [83].

### 4.3. Limitations of This Review

As a scoping review, this paper does not claim to have comprehensively identified all relevant studies on this topic but offers a structured appraisal of the current evidence base and research landscape. An alternative search strategy might include search terms specifically related to dimensions of one or more food environment frameworks. This might yield search results relating to areas under-represented or not identified through our search, such as policy conditions. The search was restricted to English language publications and did not include grey literature, including institutional reports (such as from the Food and Agriculture Organization of the United Nations, WorldFish, or the International Food Policy Research Institute), which may offer additional insights into drivers of food choice.

## 5. Conclusions

Decision making about what to eat reflects a complex set of considerations, which vary between settings, households and individuals. The use of food environment frameworks to evaluate food acquisition and consumption—considering fish within the wider diet—can provide sound evidence to guide policy and investment decisions to improve nutrition outcomes. This is particularly urgent in areas such as the AGLR, and Sub-Saharan Africa more broadly, where food and nutrition insecurity is persistent, severe and projected to increase. Identifying differential access to fish between, and within, households is essential to ensure that increases in national fish supply actually translate to nutritional benefits for the most vulnerable. Of recent food environment frameworks, variation in food access between household members is most directly articulated by the *Innocenti* Framework, which considers intra-household dynamics, eating patterns and the role of caregivers as mediators of children’s and adolescents’ diets [84]. Exploring how fish access, and diets more broadly, may differ between household members will help to address the knowledge gaps highlighted in our review.

This scoping study identifies several priorities for future research: (1) capturing temporal variation in fish acquisition and consumption, which is likely to accompany annual seasonal changes, as well as economic, environmental or political shocks that affect availability and affordability; (2) evaluating how expansion of export and urban markets and interregional trade affect fish access in lakeside and more distant communities; and (3) developing interventions, including supply increases, value chain developments or behaviour change strategies, that respond to the identified constraints of cost, availability, convenience and perceptions about quality and suitability for children. Integrated food systems research and development, with a focus on addressing constraints in rural and urban food environments, offers the potential to translate increased fish supply into improved dietary intake and nutritional status of people in the AGLR.

## Figures and Tables

**Table 1:** Number of studies providing evidence of the influence of each dimension of the food environment on fish acquisition and consumption, overall and by country.

	Dimension of Food Environment				
Number of Studies (*n*)	Availability and Physical Access	Prices and Affordability	Acceptability	Information, Guidelines and Advertising	Food Quality and Safety
Overall	14	21	17	2	5
By country:					
Kenya	6	8	9	0	4
Malawi	3	4	3	2	1
Tanzania	5	7	3	0	0
Uganda	1	0	0	0	0
Zambia	0	2	3	0	0

## Supplementary Materials

The following are available online at https://www.mdpi.com/article/10.3390/nu13072408/s1, Table S1: Evidence linking dimensions of the food environment with fish acquisition and consumption in the Great Lakes Region (studies published 2010–2021).
