# Supplementary material for: Fish Acquisition and Consumption in the African Great Lakes Region through a Food Environment Lens: A Scoping Review
Source: Nutrients. 2021 Jul 14;13(7):2408. doi: 10.3390/nu13072408 (PMC8308864; doi:10.3390/nu13072408)
Supplement: Supplementary file 1 [file nutrients-13-02408-s001.zip › nutrients-1242519-supplementary.pdf]

Table S1. Evidence linking dimensions of the food environment with fish acquisition and consumption in the Great Lakes Region (studies published 2010-2021).

Country	Location	Population	Sample size (n)	Study details	Findings on fish acquisition and consumption	Ref.
**Availability and physical access** (n = 14 studies)						
Kenya	Kitui and Vihiga sub-counties	Children 6-23 mo	335	Mixed methods study to inform food-based recommendations	Caregivers identified small whole fish as being easier to acquire than chicken.<br>Caregivers considered small whole fish to be a core food for children 6-23 mo in one of two counties; authors interpreted this to be due to proximity to Lake Victoria and several major rivers.	[1]
	Lake Naivasha	From lakeside communities (few details on methodology)	100	Questionnaire on fish consumption, in study analysing lipid content of fish species	33% respondents reported limited availability of fish in local markets, due to demand from lakeside hotels and Nairobi.<br>Respondents would consume fish more frequently if it were more available.	[2]
	Kirinyaga and Vihiga counties	Consumers in areas with many commercial fish farmers	153	Survey on consumer preferences and purchasing behaviour	25% respondents nominated ready availability as a reason for purchasing and consuming fish.<br>70% respondents reported farmed tilapia and catfish were rarely available in local markets.	[3]
	Nairobi, Nyeri, Eldoret, Nakuru and Kisumu	Urban consumers and retailers	377	Survey on consumer preferences and purchasing behaviour	Only 8% of respondents reported availability as their primary reason for consuming fish.<br>Greater availability was a reason for purchasing wild-caught rather than farmed tilapia, often directly from landing sites.	[4]
	Kisumu and Homa Bay Counties	Households in three fishing communities targeted for a study of algal blooms in Lake Victoria	88	Phone interviews to examine shifts in fish consumption, fishing activities, price changes, and coping strategies during the COVID-19 pandemic	Fishers were more likely to sell fish than consume it within their household at this stage of the COVID-19 pandemic<br>Authors suggest fishers may be targeting and selling high-value fish in order to purchase less expensive fish to consume, if they are consuming fish at all.	[5]
	Fishing communities associated with Lake Victoria (names withheld to protect confidentiality)	Male and female adults within households with a child <2 y	303 women, 253 men	Mixed methods study of transactional sexual relationships in fishing communities, including surveys and in-depth interviews	Marital relationships and other familial ties with fishers may enable women to access fish for trade or home consumption; with "fish-for-sex" relationships necessary in the absence of other relationships, or when these do not provide enough fish.<br>Acute changes in fish availability affect reliance on fish-for-sex relationships. When fish catch is high, participants described sharp increases in women's power over how they access fish.	[6]
Malawi	Machinga and Nsanje Districts, Southern Region	Children 6-36 mo in lakeside communities	355	Quantitative dietary assessments for children in a randomised controlled trial	Fish was the main source of animal protein in children's diets. Authors attribute ready availability of fish (particularly small fish) to proximity to water bodies.	[7]
	National	Nationally-representative sample (Integrated Household Survey)	12,271	Food supply data used to estimate dietary mineral supplies and prevalence of inadequate intakes	A lower risk of selenium deficiency was correlated with closer proximity to Lake Malawi; authors attributed this to greater physical access and an increased likelihood of fish consumption.	[8]
	Lilongwe and Kasungu Districts	Mothers and children less than 5 y; with different combinations of overweight / normal weight between mothers and children	274	Quantitative food frequency questionnaires to assess habitual food intake and survey on drivers of food consumption	Shorter travel time to the nearest market or shop predicted higher fish intake among mothers in the rainy season; while living in an urban area predicted intake by children in the dry season.	[9]

Country	Location	Population	Sample size (*n*)	Study details	Findings on fish acquisition and consumption	Ref.
Tanzania and Uganda	Bukoba District, Kagera Region; Kiboga District, Buganda Region	Children 12-59 mo	425	Survey to determine common dishes for children	Small dried fish were included in one of the common meals for children in Tanzania, but not in Uganda; authors attributed this to ready availability of fish due to close proximity of the Tanzanian study site to Lake Victoria.	[10]
Tanzania	Maasai Steppe, Pare Mountains, shore of Lake Victoria	Mother-child dyads from ethnic groups with differing levels of fish intake	187	Average fish intakes of three ethnic group reported (unspecified data source) in study of fatty acid status	Women of the Sengerema ethnic group, on the southern shore of Lake Victoria, ate up to 3 times that of the Pare and 5 times that of Maasai, communities that live many kilometres from any lake.	[11]
	Rufiji District, Pwani Region	Households in lakeside community	40	Mixed methods study, including monthly dietary assessments over one year	Participation in fishing was associated with greater fish consumption for all households, regardless of wealth; but this association was greatest for poor households.	[12]
					Households that caught fish ate more fish on the survey day than those that had purchased or received fish as gifts (almost twice median quantity of fish consumed).	
					Gifts were an important means of fish acquisition for female-headed households (with frequency of fish consumption nearly equivalent to male-headed households).	
	Dodoma and Morogoro Regions	Households with children 6-9 y	663	Mixed methods study to explore links between livestock-keeping and animal-source food consumption	High consumption of fish, including small fish, by households in Morogoro Region (twice as likely as in Dodoma Region); authors attribute to ready availability in markets.	[13]
	Bahi District, Dodoma Region; Mbarali District; Mbeya Region	Married men aged 29-58 y with at least two dependents	50	Focus group discussions to characterise dietary practices for men and their households	Men commonly accessed food outside the home, as well as from household meals. Fish was amongst foods consumed outside the home which would be uncommon for the wider household.	[14]
**Prices and affordability** (*n* = 15)						
Price of fish						
Kenya	Kitui and Vihiga sub-counties	Children 6-23 mo	335	Mixed methods study to inform food-based recommendations	Caregivers ranked small whole fish above chicken, in terms of affordability..	[1]
					In both study sites, small fish were sourced from markets.	
	Lake Naivasha	From lakeside communities (few details on methodology)	100	Survey on fish consumption, in study analysing lipid content of fish species	High prices are driven by demand for fish by lakeside hotels and Nairobi markets, limiting access by local households.	[2]
					37% respondents indicated cost as a barrier to consumption.	
					18% and 67% respondents would consume fish weekly and more than once per week, respectively, if prices were lower.	
	Nairobi	Low-income households with a child 1-3 y and non-pregnant woman 15-49 y	205	Survey on animal source food purchases, including expenditure, quantities, motivations and barriers	Price was the most commonly reported barrier to more frequent consumption of fish, as well as meat and eggs.	[15]
					Processed products, including dried fish, had the highest unit price of all ASFs, followed by red meat.	
					Demand for fish was most sensitive to price changes, with an 11% change in demand if price changed by 10%.	
	Kirinyaga and Vihiga counties	Consumers in areas with many commercial fish farmers	153	Survey on consumer preferences and purchasing behaviour	Few respondents indicated affordability as the reason for consuming fish.	[3]
					Only 3% respondents considered tilapia and catfish to be low-cost, compared with alternative protein sources.	
	Nairobi, Nyeri, Eldoret, Nakuru and Kisumu	Urban consumers and retailers	377	Survey on consumer preferences and purchasing behaviour	Only 3% of participants cited price as a reason for consuming tilapia and catfish.	[4]

Country	Location	Population	Sample size (*n*)	Study details	Findings on fish acquisition and consumption	Ref.
Malawi	Ntchisi District, Central Region	Local leaders, caregivers, health assistants, children 6-23 mo	Not specified	In-depth interviews observation and participatory activities	Caregivers of young children grouped fish and meat as "rare foods", consumed infrequently due to high prices.	[16]
	Blantyre and Lilongwe	Household head of person responsible for food purchases in urban households	584	Questionnaire to analyse factors influencing consumer choice and demand for two tilapia species	Price changes had a variable effect on fish purchases, with a percentage increase being associated with a reduction in quantities of fish purchased by 22% for fresh tilapia of one species (*Oreochromis shiranus*) and by 51% for a dried or smoked form of another (*Oreochromis (Nyasalapia)* spp.).	[17]
Tanzania	Bahi District, Dodoma Region	Children 6-23 mo	400	Linear programming using 24 hr dietary recalls, weighed dietary record and 7 days food records	Recommendations for optimised diets which proposed a substantial increase in the intake of small fish were associated with a two-fold increase in the overall cost of diet.	[18]
	Ukerewe Island, Lake Victoria	Individuals engaged in fishing activities, and their households	N/A	Ethnographic research on commoditisation dynamics in fishing communities	Higher quality fish is destined to international markets, and as a result changing local patterns of consumption. Fish supply chains are responding to external stakeholders at the expense of local families, with large fish such as Nile Perch unaffordable for many households.	[19]
Zambia	Lusaka District, Lusaka Province	Low-income urban households with a child 0-59 mo	714	Survey on factors influencing fish consumption	Small fish were confirmed as being affordable and accessible to poor households; authors attribute this to high availability due to the scale of capture fisheries in the region.	[20]
					Small fish are considered affordable due to their size and the ease of divisibility among family members; enabling access to large quantities for little cash outlay.	
	Lusaka District, Lusaka Province	Low-income urban households with a child 0-59 mo	714	Survey on factors influencing fish consumption	Fish consumption on the preceding day was more common in households in the highest wealth quartile, than the lowest.	[21]
					Households in the higher two wealth quartiles were more likely to consume fresh or dried forms of large fish, and to consume these fish more frequently, compared to the lower wealth quartiles.	
					There were no significant differences in the frequency of consumption of fresh small fish, dried small fish and smoked small fish between wealth groups.	
Household wealth and food security status						
Kenya	Mfangano Island, Lake Victoria	Households in a rural fishing community	111	Survey to enable modelling of associations between fishing, fish intake, and food security	Higher monthly income and household food security status were strongly associated with a higher likelihood of fish consumption.	[22]
	Kisumu and Homa Bay Counties	Households in three fishing communities targeted for a study of algal blooms in Lake Victoria	88	Phone interviews to examine shifts in fish consumption, fishing activities, price changes, and coping strategies during the COVID-19 pandemic	Frequency of fish consumption declined over three months in the early stages of the COVID-19 pandemic. Households cited access to be constrained by increasing prices (16%), reduced income (61%) and, specifically, reduced fishing income (13%).	[5]
					Higher-priced species (e.g., Nile perch and tilapia) were consumed less often, while lower-priced species (e.g., *dagaa* and cichlids) were eaten more often.	
	Fishing communities associated with Lake Victoria (names withheld to protect confidentiality)	Male and female adults within households with a child <2 y	303 women, 253 men	Mixed methods study of transactional sexual relationships in fishing communities, including surveys and in-depth interviews	Participants described "fish-for-sex" relationships as an often required extra-monetary strategy to access fish. Men and women indicated money to be insufficient to access fish for sale or home consumption, with a sexual relationship with a fisherman often necessary to make a purchase.	[6]

Country	Location	Population	Sample size (n)	Study details	Findings on fish acquisition and consumption	Ref.
Malawi	National	Nationally-representative sample (Integrated Household Survey)	12,271	Food supply data used to estimate dietary mineral supplies and prevalence of inadequate intakes	Fish consumption was associated with household expenditure quintile, with a higher probability of consumption in wealthier and urban households.	[8]
	Two rural districts in Central Malawi	Pregnant and breastfeeding women	1,230	Survey and 24 hr dietary recall to examine associations between household food insecurity and dietary diversity	Severe food insecurity was associated with a three-fold lower probability of consuming meat or fish for pregnant women, and a five-fold lower probability for breastfeeding women	[23]
	Blantyre and Lilongwe	Household head of person responsible for food purchases in urban households	584	Questionnaire to analyse factors influencing consumer choice and demand for two tilapia species	Higher household income increased the likelihood of choosing fresh tilapia over smoked or dried by 4.4% and 5.5%, for two different species; and the quantities demanded of all tilapia products by 15-22%.	[17]
					Higher education level of household food decision makers was associated with demand for greater quantities of tilapia products, but not the choice of products; authors attribute this to more education translating to more disposable income.	
Tanzania	Dodoma and Morogoro Regions	Households with children 6-9 y	663	Mixed methods study to explore links between livestock-keeping and animal-source food consumption	Market-purchased fish (including small fish) were more commonly consumed than poultry meat or eggs, despite almost three-quarters of households keeping poultry; authors suggested income from poultry-keeping may be used to purchase fish.	[13]
	Rufiji District, Pwani Region	Households in lakeside community	40	Mixed methods study, including monthly dietary assessments over one year	Rich and middle-ranked households ate freshwater fish nearly twice as often as poor households, and in greater quantities, based on mean daily consumption values.	[12]
					Richer households were more likely to preserve fish for later consumption.	
					Frequent consumption of small fish was attributed to their availability for purchase in small quantities, according to one's financial capacity.	
					Few wealthy households had cash available for higher-cost fish, and even then might by large fish on credit.	
	Kilosa District, Morogoro Region	Rural households	307	Surveys in two agricultural seasons to describe diets and evaluate links with food security	Of four dietary patterns characterised, the one with high consumption of fish (and other animal-source foods) had the lowest number of food-insecure households.	[24]
					Households with low fish consumption had increased risk of being food insecure.	
	Kongwa, Muheza and Singida Districts	Women 15-45 y involved in cultivating vegetables	252	24 hr dietary recall and survey in three non-consecutive seasons to test links between dietary diversity, vegetable production and socioeconomic status	Diets were classified diets as being of low, medium or high diversity. Fish were consumed by more than three-quarters of women with medium or high dietary diversity, compared to only one-third of those with low diversity	[25]
	Moshi Rural District, Kilimanjaro Region	Adults 15-44 y with children	1,014	Survey, food frequency questionnaire, anthropometry and micronutrient assays to test associations between food security, diets and nutritional status	Food insecurity was strongly associated with less frequent consumption of most nutrient-rich foods, including fruits and vegetables, meat and eggs; but no difference in fish consumption.	[26]
**Acceptability** (n = 9)						
Taste and other sensory attributes						
Kenya	Nairobi	Low-income households with a child 1-3 y and	205	Survey on animal source food purchases, including	Taste was the most common reason for consuming fish (as for all animal-source foods), reported by 73% households.	[15]
					A small percentage (8%) of those not consuming fish cited smell as the reason.	

Country	Location	Population	Sample size (n)	Study details	Findings on fish acquisition and consumption	Ref.
		non-pregnant woman 15-49 y		expenditure, quantities, motivations and barriers		
	Nairobi, Nyeri, Eldoret, Nakuru and Kisumu	Urban consumers and retailers	377	Survey on consumer preferences and purchasing behaviour	35% of participants considered good taste of tilapia and catfish as a reason for consumption.	[4]
					Participants reported the taste of wild-caught and farmed tilapia to differ, with some preferring wild fish and others expressing indifference.	
	Seme sub-county, Kisumu County	Adult pregnant women of known HIV status (both infected and non-infected)	49	Survey and focus group discussions to guide development and test acceptance of fish-enhanced supplemental snacks	A novel fish snack was well-accepted across four characteristics of interest. 86%, 88%, 88%, and 85% participants liked the taste, odour, texture, and colour of the fish-enhanced snacks, respectively.	[27]
	Lake Naivasha	From lakeside communities (few details on methodology)	100	Survey on fish consumption, in study analysing lipid content of fish species	10% respondents were deterred by the taste or smell of fish.	[2]
					13% respondents reported an allergy to fish.	
	Kitui and Vihiga sub-counties	Children 6-23 mo	335	Mixed methods study to inform food-based recommendations	A small number of caregivers considered small whole fish to be less appropriate for young children as they are not easily chewed. This was reflected in a lower frequency of fish consumption among children 6–11 mo, compared with children 12-23 mo.	[1]
	Kirinyaga and Vihiga counties	Consumers in areas with many commercial fish farmers	153	Survey on consumer preferences and purchasing behaviour	Authors highlight the role of taste in driving fish purchases, but this is not supported by findings of the study, in which 8% indicated taste as the reason for consuming fish, and < 4% mentioned smell as an influencing factor.	[3]
					Taste preferences varied markedly between counties, with approximately even numbers favouring tilapia (52%) and catfish (45%) in Kirinyaga, compared to a large majority favouring tilapia in Vihiga (90%). For both fish, smoked forms were the least preferred.	
	Kakamega District Hospital	Pregnant women from rural and urban areas	202	Interviews to explore dietary changes during pregnancy,including food cravings and aversions	6% women reported fish as a food craving and by 11% as a food aversion during pregnancy.	[28]
					Small fish were the most common item said to be avoided during pregnancy, by 15% of women, compared to avoidance of larger fish by 11%. This was most common during the first and early second trimester, due to nausea, vomiting, appetite loss, or aversion to taste or odour.	
Kenya and Tanzania	Shinyanga and Singida Regions, Tanzania; Elgeyo Marakwet County, Kenya	Caregivers of children 6-23 mo	Varied for different research tools	Formative research to design a behaviour change strategy to improve children's micronutrient intake, including interviews, focus group discussions, observations, market visits	Caregivers associated animal-source foods with positive attributes for children, and considered them acceptable from 6-7 mo.	[29]
					Negative perceptions of fish included fears of choking on fish bones, leading some caregivers to avoid giving fish to young children, others to take extra care in separating bones from fish.	
Malawi	Blantyre and Lilongwe	Household head of person responsible for food purchases in urban households	584	Questionnaire to analyse factors influencing consumer choice and demand for two tilapia species	Product traits were significantly associated with purchase decisions for two tilapia species under investigation. Consumers considering size were more likely to choose *Oreochromis shiranus*, while fresh *Oreochromis (Nyasalapia)* spp. were favoured for their silver colour.	[17]
					Taste had a greater influence on choice of tilapia products, than quantities purchased; with consumers favouring the taste of fresh *Oreochromis shiranus* and smoked or dried *Oreochromis (Nyasalapia)* spp.	

Country	Location	Population	Sample size (n)	Study details	Findings on fish acquisition and consumption	Ref.
	Lilongwe and Kasungu Districts	Mothers and children less than 5 y	274	Quantitative food frequency questionnaires to assess habitual food intake and survey on drivers of food consumption	Maternal taste preferences for fish predicted higher consumption of fish by women and children in the dry season. Authors identify this to be the first study to empirically measure how taste preferences predict food consumption in sub-Saharan Africa. Older child age was also associated with higher fish consumption in the dry season.	[9]
Tanzania	Rufiji District, Pwani Region	Households in lakeside community	40	Mixed methods study, including monthly dietary assessments over one year	Participants enjoyed most fish types, but there was a preference for those able to be cut into large fillets or steaks.	[12]
					Bones and viscera of smaller species were eaten, and in some cases considered a delicacy. Frying was often used to soften unpalatable tiny bones, with small fish eaten whole.	
					Women preferred oily fishes to save money on cooking oil, and those that produced a tasty broth when boiled.	
					Young children commonly receive only fish broth, while fish are served to adult men and women.	
	Ukerewe Island, Lake Victoria	Individuals engaged in fishing activities, and their households	N/A	Ethnographic research on commoditisation dynamics in fishing communities	Replacement of large fish with small fish in local diets was described as a loss made evident through tastes and senses. The enjoyment of tasting and chewing the flesh of large fish contrasted with the spikes of small fish, eaten whole or with the effort of spitting out small bones.	[19]
Zambia	Lusaka District, Lusaka Province	Low-income urban households with a child 0-59 mo	714	Survey on factors influencing fish consumption	77% respondents reported taste preference as the main reason for choosing to purchase and consume specific fish species; with no significant variation across wealth groups.	[20]
					Higher-cost tilapia was far more commonly eaten in wealthy households than poor ones; authors suggested familiarity and food habits inform poor households' acquisition of lower-cost small fish.	
	Lusaka District, Lusaka Province	Low-income urban households with a child 0-59 mo	714	Survey on factors influencing fish consumption	90% respondents indicated that there would be no change in fish consumption by women during pregnancy or lactation.	[21]
					Factors which might limit fish consumption by pregnant and lactating women were reported to include unpleasant smell; associations with evil spirits; negative effects on unborn children (e.g. being born with ringworm); rashes; vomiting and nausea.	
Convenience						
Kenya	Kitui and Vihiga sub-counties	Children 6-23 mo	335	Mixed methods study to inform food-based recommendations	Caregivers ranked small whole fish moderately, but higher than chicken, in terms of convenience as a food for young children.	[1]
Cultural factors						
Kenya	Lake Naivasha	From lakeside communities (few details on methodology)	100	Questionnaire on fish consumption, in study analysing lipid content of fish species	A small number of respondents (7%) reported a local taboo as a reason for not consuming fish, due to the skin of fish being likened to that of reptiles.	[2]
	Kajiado County	Maasai women with a child <5 y	60	Focus group discussions to investigate cultural influences on diets	Maasai culture reported to prohibit consumption of fish, chicken and wild animals. Aquatic animals viewed as unfit for human consumption, and rarely eaten.	[30]
	Kitui and Vihiga sub-counties	Children 6-23 mo	335	Mixed methods study to inform food-based recommendations	Attitudes to fish were one of the few differences in food cultures between study sites. Small fish were considered a core food for young children and households in Vihiga, with high consumption reported; but not in Kitui.	[1]
	Nairobi, Nyeri, Eldoret, Nakuru and Kisumu	Urban consumers and retailers	377	Survey on consumer preferences and purchasing behaviour	Social status was ranked lowest as a driver of tilapia and catfish consumption, reported by only 2% of households.	[4]

Country	Location	Population	Sample size (*n*)	Study details	Findings on fish acquisition and consumption	Ref.
Malawi	Lilongwe and Kasungu Districts	Mothers and children less than 5 y	55	Structured observations of food purchases in dry season and types and quantities of foods in home in rainy season; in-depth interviews on factors influencing food purchases	Respondents indicated their decisions in sourcing specific ingredients or types of food for daily meals to be shaped by Malawian food culture, in which the staple food *nsima* is accompanied by one or more side dishes of relish, often made of fish, green leafy vegetables, or beans with tomatoes and onions.	[31]
Tanzania	Rufiji District, Pwani Region	Households in lakeside community	40	Mixed methods study, including monthly dietary assessments over one year	Tradition of fishers giving fish to female relatives, romantic partners, or elderly men.	[12]
	Ukerewe Island, Lake Victoria	Individuals engaged in fishing activities, and their households	N/A	Ethnographic research on commoditisation dynamics in fishing communities	There was a widespread consensus that "fish must be on the table", regardless of what else might be eaten. Fish consumption was described as being amongst the traditional repertoire of practices for ethnic groups historically bound to the lake through fishing as a livelihood activity.	[19]
					Commoditisation of fish was implicated in causing a shift in social dynamics; from fish being eaten regularly and in large quantities, shared with neighbours; to something purchased when funds were available and eaten within the home..	
Zambia	University Teaching Hospital in Lusaka	Pregnant women attending antenatal health clinic	27	Focus group discussions to assess women's knowledge of nutrition during pregnancy and inform product development	Fish, including kapenta, were mentioned as a special food that should be consumed during pregnancy, but not specifically related to maternal, foetal or infant outcomes.	[32]
**Information, guidelines and advertising** (*n* = 1)						
Malawi	Blantyre	Children 6-59 mo, HIV-exposed and severely or moderately malnourished	36	Semi-quantitative 24 hr dietary recall and anthropometry, to assess a nutrition intervention	A two-fold increase in consumption of fish (from 22% to 47%) was observed after a six month programme including nutritional counselling and education, which advocated enriching children's porridge with small, dried or ground fish.	[33]
	Blantyre and Lilongwe	Household head of person responsible for food purchases in urban households	584	Questionnaire to analyse factors influencing consumer choice and demand for two tilapia species	Access to market information had variable effects on fish choice for different species and forms of preparation.	[17]
					Generally, consumers who access information on fish market and prices consume more tilapia products than those who do not.	
**Food quality and safety** (*n* = 5)						
Kenya	Nairobi	Low-income households with a child 1-3 y and non-pregnant woman 15-49 y	205	Survey on animal source food purchases, including expenditure, quantities, motivations and barriers	66% respondents reported nutritional value as the main reason for consuming fish (second most common reason).	[15]
					Perceived nutritional value was a common driver of animal-source food consumption, especially high for fish, milk and eggs.	
	Kitui and Vihiga sub-counties	Children 6-23 mo	335	Mixed methods study to inform food-based recommendations	Linear programming identified small whole fish as an important local source of several key nutrients (calcium, iron, zinc, vitamins B6 and B12).	[1]
					Caregivers ranked small fish lower than chicken in terms of health attributes.	
	Nairobi, Nyeri, Eldoret, Nakuru and Kisumu	Urban consumers and retailers	377	Survey on consumer preferences and purchasing behaviour	Perceptions of fish as a healthy food was most widely-cited as a reason for consuming fish (40% participants).	[4]
					Health consciousness was highest in Nairobi (48%), lowest in Kisumu (16%).	
					Some negative perceptions of farmed fish relating to health concerns about use of chemicals and genetically modified feed ingredients.	
	Kirinyaga and Vihiga counties		153	Survey on consumer preferences and purchasing behaviour	Perceptions of good quality was the main reason cited by participants for purchasing and consuming fish.	[3]

Country	Location	Population	Sample size (n)	Study details	Findings on fish acquisition and consumption	Ref.
		Consumers in areas with many commercial fish farmers			Very few participants (< 4%) attributed their consumption of fish to health attributes and nutritive value.	
Malawi	Lilongwe and Kasungu Districts	Mothers and children less than 5 y	55	Structured observations of food purchases in dry season and types and quantities of foods in home in rainy season; in-depth interviews on factors influencing food purchases	Respondents named quality of food as one of the top factors driving their food purchases. This was related to the appearance and freshness of food items, and not to food safety. Quality was mentioned in relation to all types of food, but especially fish, vegetables and fruits.	[31]

y – years of age; mo months of age

References

1. Hotz, C.; Pelto, G.; Armar-Klemesu, M.; Ferguson, E.F.; Chege, P.; Musinguzi, E. Constraints and Opportunities for Implementing Nutrition-Specific, Agricultural and Market-Based Approaches to Improve Nutrient Intake Adequacy among Infants and Young Children in Two Regions of Rural Kenya. *Matern. Child Nutr.* **2015**, *11*, 39–54, doi:10.1111/mcn.12245.
2. Keriko, J.M.; Chege, C.W.; Magu, M.M.; Mwachiro, E.C.; Murigi, A.N.; Githua, M.N.; Kareru, P.G. Fish Lipid Contents and Classes of Selected Fish Species Found in Lake Naivasha (Kenya) and the Fish Feeding Habits of the Lake's Inhabitants. *African J. Pharm. Pharmacol.* **2010**, *4*, 745–753, doi:10.5897/AJPP.9000155.
3. Obiero, K.O.; Opiyo, M.A.; Munguti, J.M.; Orina, P.S.; Kyule, D.; Yongo, E.; Muthoni Githukia, C.; Charo-Karisa, H. Consumer Preference and Marketing of Farmed Nile Tilapia (Oreochromis Niloticus) and African Catfish (Clarias Gariepinus) in Kenya: Case Study of Kirinyaga and Vihiga Counties. *Int. J. Fish. Aquat. Stud.* **2014**, *1*, 67–76.
4. Muthoni, C.; Obiero, K.; Manyala, J.; Ngugi, C.; Quagrainie, K. Consumer Perceptions and Preferences of Wild and Farmed Nile Tilapia (Oreochromis Niloticus L.) and African Catfish (Clarias Gariepinus Burchell 1822) in Urban Centres in Kenya. *Int. J. Adv. Res.* **2014**, *2*, 694–705.
5. Fiorella, K.J.; Bageant, E.R.; Mojica, L.; Obuya, J.A.; Ochieng, J.; Olela, P.; Otuo, P.W.; Onyango, H.O.; Aura, C.M.; Okronipa, H. Small-Scale Fishing Households Facing COVID-19: The Case of Lake Victoria, Kenya. *Fish. Res.* **2021**, *237*, doi:10.1016/j.fishres.2020.105856.
6. Fiorella, K.J.; Camlin, C.S.; Salmen, C.R.; Omondi, R.; Hickey, M.D.; Omollo, D.O.; Milner, E.M.; Bukusi, E.A.; Fernald, L.C.H.; Brashares, J.S. Transactional Fish-for-Sex Relationships Amid Declining Fish Access in Kenya. *World Dev.* **2015**, *74*, 323–332, doi:10.1016/j.worlddev.2015.05.015.
7. Kaimila, Y.; Divala, O.; Agapova, S.E.; Stephenson, K.B.; Thakwalakwa, C.; Trehan, I.; Manary, M.J.; Maleta, K.M. Consumption of Animal-Source Protein Is Associated with Improved Height-for-Age z Scores in Rural Malawian Children Aged 12-36 Months. *Nutrients* **2019**, *11*, doi:10.3390/nu11020480.
8. Joy, E.J.M.; Kumssa, D.B.; Broadley, M.R.; Watts, M.J.; Young, S.D.; Chilimba, A.D.C.; Ander, E.L. Dietary Mineral Supplies in Malawi: Spatial and Socioeconomic Assessment. *BMC Nutr.* **2015**, *1*, 42, doi:10.1186/s40795-015-0036-4.
9. Thakwalakwa, C.; Flax, V.L.; Phuka, J.C.; Garcia, H.; Jaacks, L.M. Drivers of Food Consumption among Overweight Mother-Child Dyads in Malawi. *PLoS One* **2020**, *15*, doi:10.1371/journal.pone.0243721.
10. Ekesa, B.; Nabuuma, D.; Kennedy, G. Content of Iron and Vitamin A in Common Foods Given to Children 12-59 Months Old from North Western Tanzania and Central Uganda. *Nutrients* **2019**, *11*, doi:10.3390/nu11030484.
11. Kuipers, R.S.; Luxwolda, M.F.; Sango, W.S.; Kwesigabo, G.; Dijck-Brouwer, D.A.J.; Muskiet, F.A.J. Maternal DHA Equilibrium during Pregnancy and Lactation Is Reached at an Erythrocyte DHA Content of 8 g/100 g Fatty Acids. *J. Nutr.* **2011**, *141*, 418–427, doi:10.3945/jn.110.128488.
12. Moreau, M.-A.; Garaway, C.J. "Fish Rescue Us from Hunger": The Contribution of Aquatic Resources to Household Food Security on the Rufiji River Floodplain, Tanzania, East Africa. *Hum. Ecol.* **2018**, *46*, 831–848, doi:10.1007/s10745-018-0030-y.
13. Bundala, N.; Kinabo, J.; Jumbe, T.; Rybak, C.; Sieber, S. Does Homestead Livestock Production and Ownership Contribute to Consumption of Animal Source Foods? A Pre-Intervention Assessment of Rural Farming Communities in Tanzania. *Sci. African* **2020**, *7*, e00252, doi:10.1016/j.sciaf.2019.e00252.
14. Ochieng, J.; Afari-Sefa, V.; Lukumay, P.J.; Dubois, T. Determinants of Dietary Diversity and the Potential Role of Men in Improving Household Nutrition in Tanzania. *PLoS One* **2017**, *12*, doi:10.1371/journal.pone.0189022.
15. Cornelsen, L.; Alarcon, P.; Häsler, B.; Amendah, D.D.; Ferguson, E.; Fèvre, E.M.; Grace, D.; Dominguez-Salas, P.; Rushton, J. Cross-Sectional Study of Drivers of Animal-Source Food

Consumption in Low-Income Urban Areas of Nairobi, Kenya. *BMC Nutr.* **2016**, *2*, 70, doi:10.1186/s40795-016-0109-z.
16. Kodish, S.; Aburto, N.; Hambayi, M.N.; Kennedy, C.; Gittelsohn, J. Identifying the Sociocultural Barriers and Facilitating Factors to Nutrition-Related Behavior Change: Formative Research for a Stunting Prevention Program in Ntchisi, Malawi. *Food Nutr. Bull.* **2015**, *36*, 138–153, doi:10.1177/0379572115586784.
17. Chikowi, C.T.M.; Ochieng, D.O.; Jumbe, C.B.L. Consumer Choices and Demand for Tilapia in Urban Malawi: What Are the Complementarities and Trade-Offs? *Aquaculture* **2021**, *530*, doi:10.1016/j.aquaculture.2020.735755.
18. Raymond, J.; Agaba, M.; Mollay, C.; Rose, J.W.; Kassim, N. Analysis of Nutritional Adequacy of Local Foods for Meeting Dietary Requirements of Children Aged 6-23 Months in Rural Central Tanzania. *Arch. Public Heal.* **2017**, *75*, 8, doi:10.1186/s13690-017-0226-4.
19. Allegretti, A. "We Are Here to Make Money": New Terrains of Identity and Community in Small-Scale Fisheries in Lake Victoria, Tanzania. *J. Rural Stud.* **2019**, *70*, 49–57, doi:10.1016/j.jrurstud.2019.05.006.
20. Genschick, S.; Marinda, P.; Tembo, G.; Kaminski, A.M.; Thilsted, S.H. Fish Consumption in Urban Lusaka: The Need for Aquaculture to Improve Targeting of the Poor. *Aquaculture* **2018**, *492*, 280–289, doi:10.1016/j.aquaculture.2018.03.052.
21. Marinda, P.A.; Genschick, S.; Khayeka-Wandabwa, C.; Kiwanuka-Lubinda, R.; Thilsted, S.H. Dietary Diversity Determinants and Contribution of Fish to Maternal and Under-Five Nutritional Status in Zambia. *PLoS One* **2018**, *13*, 18, doi:10.1371/journal.pone.0204009.
22. Fiorella, K.J.; Hickey, M.D.; Salmen, C.R.; Nagata, J.M.; Mattah, B.; Magerenge, R.; Cohen, C.R.; Bukusi, E.A.; Brashares, J.S.; Fernald, L.H. Fishing for Food? Analyzing Links between Fishing Livelihoods and Food Security around Lake Victoria, Kenya. *Food Secur.* **2014**, *6*, 851–860, doi:10.1007/s12571-014-0393-x.
23. Kang, Y.; Hurley, K.M.; Ruel-Bergeron, J.; Monclus, A.B.; Oemcke, R.; Wu, L.S.F.; Mitra, M.; Phuka, J.; Klemm, R.; West Jr., K.P.; et al. Household Food Insecurity Is Associated with Low Dietary Diversity among Pregnant and Lactating Women in Rural Malawi. *Public Health Nutr.* **2019**, *22*, 697–705, doi:10.1017/s1368980018002719.
24. Ntwenya, J.E.; Msuya, J.; Mamiro, P.; Kinabo, J.; Majili, Z.S. Correlates of the Dietary Patterns Factor Loadings Scores during Rainy Season among 307 Subjects Studied, Adult Rural Household Members from the Kilosa District, Tanzania. *Figshare* **2015**, doi:http://dx.doi.org/10.1371/journal.pone.0126038.t011.
25. Keding, G.B.; Msuya, J.M.; Maass, B.L.; Krawinkel, M.B. Relating Dietary Diversity and Food Variety Scores to Vegetable Production and Socio-Economic Status of Women in Rural Tanzania. *Food Secur.* **2012**, *4*, 129–140, doi:10.1007/s12571-011-0163-y.
26. Leyna, G.H.; Mmbaga, E.J.; Mnyika, K.S.; Hussain, A.; Klepp, K.I. Food Insecurity Is Associated with Food Consumption Patterns and Anthropometric Measures but Not Serum Micronutrient Levels in Adults in Rural Tanzania. *Public Health Nutr.* **2010**, *13*, 1438–1444, doi:10.1017/s1368980010000327.
27. Gewa, C.A.; Frankenfeld, C.L.; Slavin, M.; Omondi, M. Fish-Enhanced and Soybean-Enhanced Supplemental Snacks Are Acceptable among Pregnant Women in Rural Kenya. *Food Nutr. Bull.* **2014**, *35*, S180–S187, doi:10.1177/15648265140354s303.
28. Kariuki, L.; Lambert, C.; Purwestri, R.C.; Maundu, P.; Biesalski, H.K. Role of Food Taboos in Energy, Macro and Micronutrient Intake of Pregnant Women in Western Kenya. *Nutr. Food Sci.* **2017**, *47*, 795–807, doi:10.1108/nfs-09-2016-0146.
29. Robert, R.C.; Bartolini, R.M.; Creed-Kanashiro, H.M.; Verney Sward, A. Using Formative Research to Design Context-Specific Animal Source Food and Multiple Micronutrient Powder Interventions to Improve the Consumption of Micronutrients by Infants and Young Children in Tanzania, Kenya, Bangladesh and Pakistan. *Matern. Child Nutr.* **2021**, *17*, doi:10.1111/mcn.13084.
30. Chege, P.M.; Kimiywe, J.O.; Ndungu, Z.W. Influence of Culture on Dietary Practices of Children under Five Years among Maasai Pastoralists in Kajiado, Kenya. *Int. J. Behav. Nutr. Phys. Act.* **2015**, *12*, doi:10.1186/s12966-015-0284-3.
31. Flax, V.L.; Thakwalakwa, C.; Schnefke, C.H.; Phuka, J.C.; Jaacks, L.M. Food Purchasing Decisions of Malawian Mothers with Young Children in Households Experiencing the Nutrition Transition. *Appetite* **2021**, *156*, doi:10.1016/j.appet.2020.104855.
32. Chunda-Liyoka, C.; Lubeya, M.K.; Imakando, M.; Kisling, S.; Majid, S.; Willis, M.S.; Wood, C.; Kankasa, C.; DiRusso, C.C. Healthy Pregnancies and Essential Fats: Focus Group Discussions with Zambian Women on Dietary Need and Acceptability of a Novel RUSF Containing Fish Oil DHA. *BMC Pregnancy Childbirth* **2020**, *20*, 14, doi:10.1186/s12884-020-2783-8.
33. Buonomo, E.; de Luca, S.; Tembo, D.; Scarcella, P.; Germano, P.; Altan, A.M.D.; Palombi, L.; Liotta, G.; Nielsen-Saines, K.; Erba, F.; et al. Nutritional Rehabilitation of HIV-Exposed Infants in Malawi: Results from the Drug Resources Enhancement Against AIDS and Malnutrition Program. *Int. J. Environ. Res. Public Health* **2012**, *9*, 421–434, doi:10.3390/ijerph9020421.
